# A Multi-Branch Network for Integrating Spatial, Spectral, and Temporal Features in Motor Imagery EEG Classification

**DOI:** 10.3390/brainsci15080877

**Published:** 2025-08-18

**Authors:** Xiaoqin Lian, Chunquan Liu, Chao Gao, Ziqian Deng, Wenyang Guan, Yonggang Gong

**Affiliations:** 1 School of Computer and Artificial Intelligence, Beijing Technology and Business University, Beijing 102488, China; lianxq@th.btbu.edu.cn (X.L.); 2330602060@st.btbu.edu.cn (C.L.); deng_zq@st.btbu.edu.cn (Z.D.); 20151005@btbu.edu.cn (W.G.); gongyg@btbu.edu.cn (Y.G.); 2 Key Laboratory of Industrial Internet and Big Data, China National Light Industry, Beijing Technology and Business University, Beijing 102488, China

**Keywords:** brain-computer interface, electroencephalogram, multi-class motor imagery, power spectral density, convolutional neural network, depthwise separable convolution

## Abstract

**Background**: Efficient decoding of motor imagery (MI) electroencephalogram (EEG) signals is essential for the precise control and practical deployment of brain-computer interface (BCI) systems. Owing to the complex nonlinear characteristics of EEG signals across spatial, spectral, and temporal dimensions, efficiently extracting multidimensional discriminative features remains a key challenge to improving MI-EEG decoding performance. **Methods**: To address the challenge of capturing complex spatial, spectral, and temporal features in MI-EEG signals, this study proposes a multi-branch deep neural network, which jointly models these dimensions to enhance classification performance. The network takes as inputs both a three-dimensional power spectral density tensor and two-dimensional time-domain EEG signals and incorporates four complementary feature extraction branches to capture spatial, spectral, spatial-spectral joint, and temporal dynamic features, thereby enabling unified multidimensional modeling. The model was comprehensively evaluated on two widely used public MI-EEG datasets: EEG Motor Movement/Imagery Database (EEGMMIDB) and BCI Competition IV Dataset 2a (BCIIV2A). To further assess interpretability, gradient-weighted class activation mapping (Grad-CAM) was employed to visualize the spatial and spectral features prioritized by the model. **Results**: On the EEGMMIDB dataset, it achieved an average classification accuracy of 86.34% and a kappa coefficient of 0.829 in the five-class task. On the BCIIV2A dataset, it reached an accuracy of 83.43% and a kappa coefficient of 0.779 in the four-class task. **Conclusions**: These results demonstrate that the network outperforms existing state-of-the-art methods in classification performance. Furthermore, Grad-CAM visualizations identified the key spatial channels and frequency bands attended to by the model, supporting its neurophysiological interpretability.

## 1. Introduction

Brain-computer interfaces (BCIs), a promising technology for neural interfacing, have attracted substantial research interest in recent years. The primary objective of BCIs is to establish effective communication between the human brain and external devices, enabling control of computers, prosthetic limbs, and robots through brain electrical activity [1]. Among brain signal acquisition modalities, electroencephalography (EEG) has emerged as one of the most widely used in modern BCI systems due to its ease of acquisition, low cost, and high temporal resolution. EEG-based BCIs show broad application potential in rehabilitation medicine, assistive device control, and virtual reality [2].

Among BCI paradigms, motor imagery (MI) has been extensively studied. MI involves eliciting neural activity in motor-related brain regions by imagining a movement without actual execution [3]. Compared with other BCIs, MI-based BCIs offer the advantage of not requiring physical movement. Even individuals with motor impairments can generate MI-EEG signals, enabling control of external devices purely through neural intent. This capability not only provides an effective communication pathway for people with disabilities but also promotes wider adoption and personalization of BCI technology [4]. Advancing MI-EEG decoding methods can further improve the accuracy and robustness of MI-BCI systems, thus advancing their practical deployment.

Researchers have extensively investigated MI-EEG decoding, leading to a variety of effective approaches that generally fall into two categories: traditional machine learning and deep learning [5].

Traditional machine learning methods typically combine handcrafted feature extraction techniques with conventional classifiers. Among them, the common spatial pattern (CSP) algorithm [6] is widely used for MI-EEG classification. CSP learns spatial filters that maximize inter-class variance while minimizing intra-class variance, thereby extracting discriminative spatial features from EEG data [7]. However, CSP has several limitations, motivating researchers to propose various enhancements [8]. For example, to address its limitations in frequency-band selection, the filter bank common spatial pattern (FBCSP) algorithm [9] extends the CSP by extracting features across multiple frequency bands, thereby improving the adaptability and classification accuracy. Another major limitation of CSP is its sensitivity to noise and the scarcity of training data. The regularized common spatial pattern (RCSP) algorithm [10] addresses this issue by incorporating prior information or penalty terms into the optimization, thereby enhancing robustness and generalization.

Regarding classifiers, traditional algorithms such as support vector machines (SVM) [11] and linear discriminant analysis (LDA) [12] are commonly used. Although these classifiers can achieve satisfactory performance, they rely on manual feature selection and parameter tuning and often fail to capture the complex nonlinear characteristics of EEG signals [5]. As EEG decoding tasks grow more complex, the inability of traditional machine learning methods to handle high-dimensional data and represent intricate features becomes increasingly evident, ultimately limiting the decoding accuracy.

With the rapid development of deep learning, deep neural networks have been increasingly used for EEG decoding to overcome these limitations in modeling high-dimensional and nonlinear data. Deep learning techniques, particularly convolutional neural networks (CNNs), automatically extract hierarchical feature representations through multi-layer nonlinear transformations, thereby enhancing EEG classification performance [13]. For example, Deep ConvNet and Shallow ConvNet, proposed by Schirrmeister et al. [14], are representative early deep learning models for EEG decoding. Deep ConvNet comprises five convolutional layers and learns multi-level spatiotemporal features, offering strong generalization ability. In contrast, Shallow ConvNet contains two convolutional layers and applies square and logarithmic nonlinearities at the output, specifically designed to extract frequency-band energy features from oscillatory EEG signals. These networks have demonstrated strong EEG classification performance by effectively capturing both spatiotemporal and spectral patterns. However, their reliance on multiple layers of conventional 2D convolution results in large parameter counts, increased computational and memory demands, and slower inference, hindering real-time or resource-limited deployment.

To address these issues, depthwise separable convolution (DSC) has been introduced as an architectural optimization, factorizing conventional convolutions into depthwise and pointwise operations to reduce both model complexity and computational cost. This advantage is particularly beneficial for high-dimensional EEG data, enabling more efficient feature extraction and faster model training. EEGNet, proposed by Lawhern et al. [15], was the first model to adopt DSC for EEG classification, reducing the parameters from over 100,000 in Deep ConvNet to only a few thousand, while maintaining competitive accuracy. Although this compression may limit the network’s ability to capture deeper semantic information, EEGNet remains effective due to its compact and efficient design, making it suitable for resource-constrained environments.

Recently, several MI-EEG classification networks incorporating DSC have shown promising performance. For example, AMSI-EEGNet [16] employs a multi-scale input strategy with integrated DSC modules, achieving an average accuracy of 76.27% in four-class MI classification on the BCI Competition IV 2a (BCIIV2A) dataset, while significantly reducing the computational complexity. Yu et al. [17] proposed MBCNN-TCN-Net, which combines a multi-branch CNN architecture with temporal convolutional network (TCN) modules to improve the temporal feature representation, achieving 75.08% accuracy on the same dataset. Qin et al. [18] introduced M-FANet, integrating multiple attention mechanisms with DSC to extract spectral, spatial, and feature-map-level features, achieving 77.86% accuracy on the same dataset. Collectively, these studies demonstrate that DSC reduces the computational cost while maintaining reliable classification performance in MI-EEG decoding tasks.

Building on DSC-based architectures, BrainGridNet [19] effectively extracts spatial features from three-dimensional power spectral density (3D PSD) matrices using a dual-branch design. However, it lacks an explicit mechanism for frequency-specific feature extraction. This limitation hinders its ability to capture discriminative spectral patterns across frequency bands, which are critical for MI-EEG classification. To address this, this study extends BrainGridNet by adding two new branches: a spectral feature extraction branch and a spatial-spectral joint feature extraction branch, aiming to improve classification performance in multi-class MI tasks.

However, relying solely on 3D PSD-based features overlooks the temporal dynamics of EEG signals, which are crucial for capturing the evolution of brain activity over time. Motivated by AMSI-EEGNet [16] and the need to capture temporal structure, this study incorporates a temporal feature extraction branch to model time-domain characteristics, enriching the model’s representational capacity.

This study aims to develop a multi-branch neural network, referred to as MSSTNet, to jointly model spatial, spectral, and temporal characteristics of MI-EEG signals for comprehensive multi-feature representation and accurate classification. Furthermore, this study aims to investigate the neural interpretability of the proposed model to validate its physiological relevance and shed light on underlying neural mechanisms. The specific objectives are as follows:

(1) To extract spatial and spectral features from EEG signals, a 3D PSD tensor is constructed as the primary network input. To compensate for the absence of temporal dynamics in the 3D PSD representation, 2D time-domain EEG signals are introduced as a complementary input.
(2) To capture spatial, spectral, spatial-spectral joint, and temporal features of MI-EEG signals, while maintaining low computational complexity, four parallel feature extraction branches based on DSC modules are designed.
(3) To evaluate the classification performance of the proposed model, comparative experiments are conducted on two publicly available MI-EEG datasets, benchmarking against state-of-the-art methods.
(4) To enhance the model’s neurophysiological interpretability, gradient-weighted class activation mapping (Grad-CAM) is applied to visualize the brain regions and frequency bands most relevant to the classification tasks.

## 2. Materials and Methods

### 2.1. Dataset and Preprocessing

This study uses two publicly available MI-EEG datasets for model training and performance evaluation.

#### 2.1.1. EEG Motor Movement/Imagery Database (EEGMMIDB)

The EEGMMIDB dataset [20] contains MI-EEG recordings from 109 healthy subjects. The EEG data were acquired with 64 electrodes placed according to the international 10-20 system, at a sampling rate of 160 Hz. Each subject completed 14 experimental sessions: R01 and R02 correspond to eyes-open and eyes-closed resting tasks, respectively; R03, R07, and R11 are motor execution tasks involving repeated opening and closing of the left or right hand; R04, R08, and R12 are MI tasks involving imagined opening and closing of the left or right hand; R05, R09, and R13 are motor execution tasks involving repeated opening and closing of both hands or both feet; and R06, R10, and R14 are MI tasks involving imagined opening and closing of both hands or both feet.

**Figure 1:**
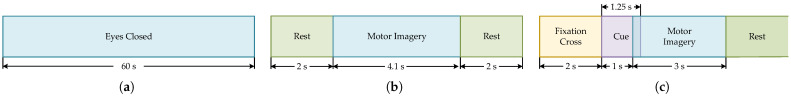
Experimental paradigms for the two datasets (“s” denotes seconds). (**a**) The paradigm of an eyes-closed trial on the EEGMMIDB dataset. (**b**) The paradigm of an MI trial on the EEGMMIDB dataset. (**c**) The paradigm of an MI trial on the BCIIV2A dataset.

Data from sessions R02, R04, R06, R08, R10, R12, and R14 of the first 10 subjects were used, covering the eyes-closed resting state and four MI task types. The experimental paradigm for the eyes-closed resting task is illustrated in Figure 1a, with an original recording duration of 60 s. To construct training samples, 60 s of continuous recording were segmented into 4.1 s windows with a 2.7 s step, yielding 21 samples from the R02 session. The experimental paradigm for the MI tasks is presented in Figure 1b. Each trial lasted 4.1 s, with 21 trials selected for each of the four MI task types: left hand, right hand, both hands, and both feet. This resulted in a five-class problem, including the eyes-closed resting state and the four MI categories.

#### 2.1.2. BCI Competition IV Dataset 2a (BCIIV2A)

The BCIIV2A dataset [21] contains MI-EEG data from 9 healthy subjects performing four MI tasks: imagining movements of the left hand, right hand, feet, and tongue. The EEG data were acquired with 25 electrode, including three dedicated to electrooculogram (EOG) recording, following the international 10-20 system, at a sampling rate of 250 Hz. The experimental paradigm of this dataset is illustrated in Figure 1c. Each trial began with a 2 s fixation, followed by a 1.25 s cue. Subjects initiated the MI task 1 s after cue onset and maintained the imagery for 3 s, followed by a rest interval before the next trial. Each MI class included 72 trials, yielding 288 trials per subject for model training and evaluation.

#### 2.1.3. Data Preprocessing

To ensure consistency across datasets and improve both learning efficiency and generalization, this study applied a unified preprocessing pipeline to the raw EEG signals, consisting of the following steps:

(1) Baseline Correction

Baseline correction was applied to remove artifacts caused by equipment drift, power-line interference, and other low-frequency noise. For each EEG segment, the 1 s pre-event resting-state signal served as the baseline, and its mean potential was subtracted from each channel. Note that for the eyes-closed samples in the EEGMMIDB dataset, baseline correction was not applied.

(2) Sampling Rate Alignment

Because the EEGMMIDB dataset was sampled at 160 Hz and the BCIIV2A dataset at 250 Hz, the latter was downsampled to 160 Hz for uniform input dimensionality. Direct downsampling without preprocessing can cause frequency components above the Nyquist frequency (80 Hz) to alias into lower bands, introducing spurious low-frequency information and degrading signal quality [22]. To prevent aliasing, this study applied a low-pass anti-aliasing filter (cutoff: 80 Hz) before downsampling, thereby preserving the signal quality and feature integrity.

(3) Amplitude Conversion and Standardization

All EEG signals were multiplied by $10^{6}$ to convert from volts (V) to microvolts (μV), matching the range required by the neural network inputs. This study then applied Z-score normalization to each sample to mitigate inter-subject and inter-channel amplitude variability, ensuring consistent scaling for stable training and better generalization. The z-score normalization is defined as:

(1) $$\hat{x}\left(t\right)=\frac{x(t)-\mu }{\sigma },$$

where x(t) denotes the EEG signal of a given channel before standardization; $\hat{x}\left(t\right)$ denotes the standardized EEG signal of the same channel; μ represents the mean value of the original EEG signal; and σ denotes the standard deviation.

(4) Sliding Window Segmentation

A sliding window strategy was used to augment the datasets and improve generalization. For both datasets, a 1.5 s window with a 0.2 s step was applied to the standardized EEG signals, generating overlapping segments to increase the sample diversity and support more effective feature learning and classification.

### 2.2. Three-Dimensional Power Spectral Density Matrix

Three-dimensional EEG tensor representations often yield superior decoding performance compared with conventional two-dimensional forms [23]. To preserve both spatial and spectral features, this study constructs a three-dimensional power spectral density (3D PSD) matrix as input to the proposed network.

**Figure 2:**
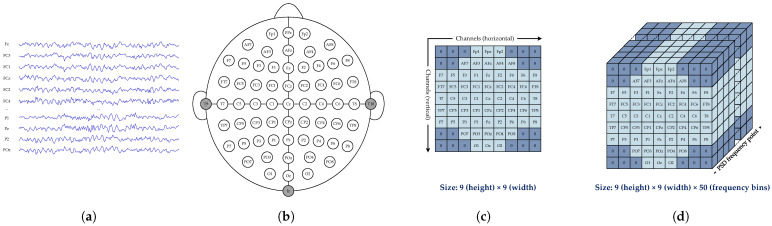
Construction method of 3D PSD matrix. (**a**) EEG signal waveforms. (**b**) The layout of 64 EEG electrodes based on the international 10-20 system. (**c**) Two-dimensional electrode topological map. (**d**) Three-dimensional PSD matrix.

Figure 2a shows the preprocessed EEG signals. For each EEG channel, the PSD was estimated using Welch’s method [24] over the frequency range 0–50 Hz at 1 Hz resolution, producing a spectral vector with a length of 50. The PSD vector for channel *c* is given by

(2) $$PSD_{c}=\left[P_{c}\left(f_{1}\right),P_{c}\left(f_{2}\right),\ldots ,P_{c}\left(f_{k}\right),\ldots ,P_{c}\left(f_{50}\right)\right],\;f_{k}=k\;\mathrm{Hz},\;k=1,2,\ldots ,50,$$

where $PSD_{c}$ denotes the PSD vector of channel *c*, and $P_{c}\left(f_{k}\right)$ is the PSD value of channel *c* at frequency $f_{k}$.

Figure 2b illustrates the 64-electrode layout based on the international 10-20 system. To preserve the spatial structure, these electrodes were mapped to a 9×9 topological grid according to their spatial coordinates, as shown in Figure 2c. Empty positions were zero-padded, and the PSD vector $PSD_{c}$ corresponding to each electrode was sequentially assigned to its grid position. Through this process, a 3D PSD tensor is constructed, as shown in Figure 2d. This tensor integrates the spatial configuration and spectral characteristics of EEG signals, providing a structured input for the model to learn cross-dimensional representations.

### 2.3. Depthwise Separable Convolution

Compared with conventional CNNs, employing depthwise separable convolution (DSC) substantially reduces model parameters and computational cost while maintaining robust feature extraction capability [25]. Consequently, DSC is used in this study as a core component of the proposed network architecture.

**Figure 3:**
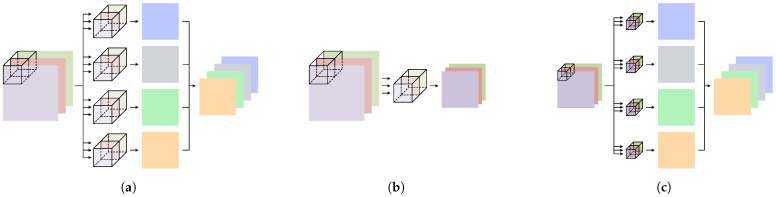
Schematic diagrams of a 2D standard convolution, a 2D depthwise convolution, and a 2D pointwise convolution. (**a**) A 2D standard convolution requires $C_{\mathrm{out}}$ convolutional kernels of size $K\times K\times C_{\mathrm{in}}$. (**b**) A 2D depthwise convolution requires 1 convolutional kernel of size $K\times K\times C_{\mathrm{in}}$. (**c**) A 2D pointwise convolution requires $C_{\mathrm{out}}$ convolutional kernels of size $1\times 1\times C_{\mathrm{in}}$.

Figure 3a illustrates a schematic diagram of a 2D standard convolution in conventional CNNs. For an input feature map of size $H\times W\times C_{\mathrm{in}}$, producing an output with $C_{\mathrm{out}}$ channels requires a kernel of size $K\times K\times C_{\mathrm{in}}$ for each output channel. Each kernel performs a weighted sum across all input channels, enabling simultaneous spatial feature extraction and cross-channel fusion. The total number of parameters in a 2D standard convolution is given by

(3) $$Paras_{2D\_\mathrm{SC}}=K\times K\times C_{\mathrm{in}}\times C_{\mathrm{out}}.$$

Although standard convolutions offer strong expressive power, their parameters and computational cost grow rapidly with increasing channel count and kernel size. This may lead to overfitting and slow inference, especially in deep architectures. To address these challenges, DSC is applied to reduce model complexity while preserving high feature-representation capacity. DSC decomposes into two independent operations: depthwise convolution and pointwise convolution.

In 2D depthwise convolution, each input channel is assigned an independent K×K kernel, which performs spatial convolution within that channel without cross-channel interaction. Figure 3b illustrates a schematic diagram of a 2D depthwise convolution. For input size $H\times W\times C_{\mathrm{in}}$, the number of output channels remains $C_{\mathrm{in}}$, and the parameter count is given by

(4) $$Paras_{2D\_\mathrm{DC}}=K\times K\times C_{\mathrm{in}}.$$

Compared to 2D standard convolution, 2D depthwise convolution greatly reduces parameters and computation but lacks cross-channel information fusion, limiting expressive power. To overcome this, pointwise convolution is applied.

Two-dimensional pointwise convolution is essentially a 1 × 1 convolution that weights and combines information across different channels, enabling cross-channel fusion and recombination [26]. Figure 3c illustrates a schematic diagram of a 2D pointwise convolution. For input size $H\times W\times C_{\mathrm{in}}$, it uses $C_{\mathrm{out}}$ kernels of size $1\times 1\times C_{\mathrm{in}}$, yielding an output with $C_{\mathrm{out}}$ channels. The parameter count is given by

(5) $$Paras_{2D\_\mathrm{PC}}=C_{\mathrm{in}}\times C_{\mathrm{out}}.$$

Since pointwise convolution does not increase the computational complexity and enables cross-channel interaction, it is typically combined with depthwise convolution to form the DSC. The parameter count of the 2D DSC is

(6) $$Paras_{2D\_\mathrm{DSC}}=C_{\mathrm{in}}\times \left(K\times K+C_{\mathrm{out}}\right)$$

Comparing Equations (3) and (6) under identical input and output dimensions shows that the 2D DSC can drastically reduce the parameters and computation. For example, with K=3 and $C_{\mathrm{in}}=C_{\mathrm{out}}=64$, 2D DSC requires only about 1/8 of the parameters of 2D standard convolution, significantly lowering the memory usage and computational cost.

By combining depthwise convolution for capturing local spatial features with pointwise convolution for cross-channel fusion, DSC retains strong feature extraction capability. As a result, it has been widely adopted in lightweight networks such as MobileNet [27] to improve the deployment efficiency and training speed. In this study, DSC modules are integrated into multiple feature extraction branches of MSSTNet to balance the expressive power and computational efficiency, enhancing the performance and generalization in resource-constrained scenarios.

### 2.4. Network Architecture

**Figure 4:**
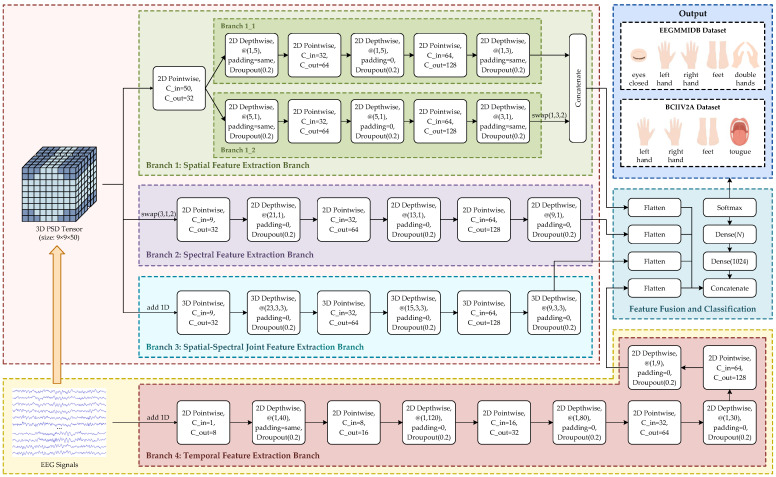
The overall framework of the proposed MSSTNet model. ($C_{\mathrm{in}}$ denotes the numbers of input channels of a convolutional module; $C_{\mathrm{out}}$ denotes the numbers of output channels of a convolutional module. *N* denotes the number of output categories).

To fully exploit EEG signals characteristics across spatial, spectral, and temporal domains, MSSTNet adopts a multi-branch convolutional architecture for feature extraction. The overall structure of MSSTNet is shown in Figure 4. It comprises four parallel branches, each dedicated to modeling spatial, spectral, spatial-spectral joint, and temporal features. The outputs from these branches are fused in deeper layers, and classification is performed via a fully connected layer.

**Figure 5:**
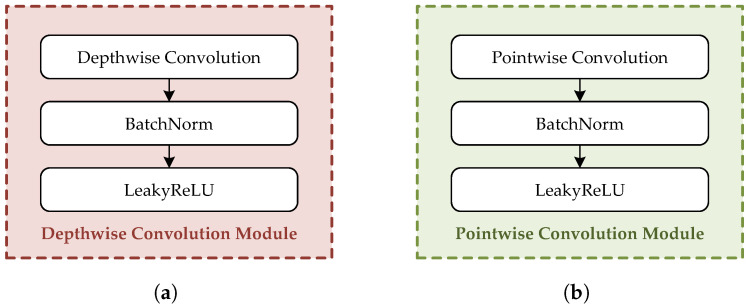
The structures of DSC submodules. (**a**) Depthwise convolution module. (**b**) Pointwise convolution module.

Each branch employs DSC to reduce the parameter count and improve the computational efficiency. As illustrated in Figure 5, each DSC submodule integrates batch normalization and the LeakyReLU activation function to ensure training stability and nonlinear expressiveness. This unified design enhances the consistency of feature extraction and facilitates deployment in resource-constrained environments.

The parameter settings for each block are detailed in the branch descriptions below and have been experimentally tuned to ensure consistent output dimensions, enabling effective feature fusion and classification. This study next describes the structural design and modeling objectives of each branch.

#### 2.4.1. Branch 1: Spatial Feature Extraction Branch

Branch 1 takes the 3D PSD tensor as input and is designed to capture the spatial distribution of EEG activity across electrode positions. It incorporates two orthogonal convolution paths to extract spatial features along the longitudinal and lateral electrode dimensions. Before these paths, a pointwise convolution module compresses the spectral dimension from 50 to 32 channels. This reduces the parameter and computational cost while mitigating redundancy in spectral information, thus facilitating the extraction of discriminative spatial features downstream.

In the vertical path (Branch 1_1), a 1×5 depthwise convolution first captures long-range dependencies between electrodes along the vertical axis. A pointwise convolution then integrates channel information, followed by a 1×3 depthwise convolution to refine local spatial features. This two-stage design enables the modeling of both global and local dependencies. In the horizontal path (Branch 1_2), a series of 1×N kernels with progressively decreasing width capture lateral dependencies from broad to narrow spatial scales. Finally, the outputs of both paths are concatenated along the channel dimension, yielding a bidirectional spatial representation that integrates vertical and horizontal structures, providing a comprehensive and discriminative spatial feature set for classification.

#### 2.4.2. Branch 2: Spectral Feature Extraction Branch

Branch 2 is designed to capture the spectral characteristics of EEG signals. In contrast to Branch 1, this branch analyzes variations in EEG signals along the spectral axis. It extracts correlations between different frequency points to identify key spectral features that reflect neural oscillatory activity, providing discriminative information for MI classification.

To achieve effective spectral domain modeling, this branch first rearranges the dimensions of the 3D PSD tensor, aligning the spectral axis as the primary convolutional direction, thereby enabling subsequent convolutions to focus on spectral features. A 2D pointwise convolution module is then applied to expand the channel dimension from 9 to 32, increasing the resolution for subsequent convolutions and enhancing the model’s ability to learn discriminative spectral features.

This branch sequentially applies 2D depthwise convolution modules with kernel sizes of 21×1, 13×1, and 9×1, corresponding to large, medium, and small receptive fields. These modules extract global spectral structures, local band features, and fine-grained details, respectively. This multi-scale design helps construct a hierarchical feature representation, enabling the network to capture neural activity differences across various frequency bands. These convolutions operate exclusively along the spectral dimension, ensuring that spectral feature modeling remains independent from spatial structures and enhancing the model’s interpretability of spectral responses.

#### 2.4.3. Branch 3: Spatial-Spectral Joint Feature Extraction Branch

While 2D DSC is effective for feature modeling, it typically only focuses on either the spatial or spectral dimension. To better capture the coupled relationships between EEG signals across both spatial and spectral dimensions, Branch 3 introduces an extended form of 2D DSC: 3D DSC [28]. The 3D DSC combines depthwise and pointwise convolutions in three dimensions, enabling independent modeling along the spectral axis, horizontal spatial axis, and vertical spatial axis. This approach allows for joint multi-dimensional feature extraction while maintaining parameter efficiency.

This branch takes the 3D PSD tensor as input and first applies a 3D pointwise convolution module to compress and map the input feature channels, preparing the data for subsequent 3D DSC processing. The branch then stacks three 3D depthwise convolution modules with progressively smaller kernel sizes: 23×3×3, 15×3×3, and 9×3×3. The larger kernels capture cross-frequency band spectral joint features, improving the sensitivity to the co-occurrence of low- and high-frequency activity. Meanwhile, the smaller kernels in the spatial dimensions preserve local resolution and refine electrode coupling modeling. Each of the first two 3D depthwise convolution modules is followed by a 3D pointwise convolution module for cross-channel information fusion, while the final 3D depthwise convolution module omits the pointwise convolution module to reduce model complexity and avoid redundant computations.

Through this process of layer-wise abstraction and compression, Branch 3 creates a multi-scale representation of the spectral-spatial coupling structure, effectively capturing the space-frequency pattern variations induced by the co-activation of brain regions.

#### 2.4.4. Branch 4: Temporal Feature Extraction Branch

Although the 3D PSD tensor effectively integrates the spatial and spectral features of EEG signals, it does not capture the temporal dynamics of neural activity. To address this gap, Branch 4 takes the 2D time-domain EEG signals as input and focuses on modeling the dynamic changes in neural activity over time.

Initially, a 2D pointwise convolution module is applied to expand the single input channel into 9 channels, creating a multi-channel feature representation space that provides a richer foundation for subsequent temporal modeling. The model then alternately stacks five 2D depthwise convolution modules and four 2D pointwise convolution modules, progressively enhancing its ability to capture features across different time scales.

In this branch, all 2D depthwise convolution operations use 1×K kernels and operate exclusively along the time dimension, avoiding the mixing of information across channels and focusing on extracting temporal features. To capture global dynamic features while preserving time boundary information, the first convolution stage introduces a depthwise convolution module with a kernel size of 1×40, combined with symmetric padding. This ensures that the output length remains unchanged while obtaining a wide receptive field for global dynamic information. Subsequently, the branch applies four depthwise convolution modules with kernel sizes of 1×120, 1×80, 1×30, and 1×9, creating a multi-scale time window structure that covers both long-term and short-term time scales. This coarse-to-fine feature extraction process enables the model to capture long-term neural activity trends and short-term dynamic changes, thereby improving its ability to recognize key patterns in non-stationary MI signals.

To mitigate overfitting in high-dimensional feature spaces, MSSTNet incorporates dropout layers at appropriate points in each of its four feature extraction branches. This regularization mechanism effectively suppresses feature redundancy and enhances the model’s generalization ability [29].

Finally, the four branches of MSSTNet output feature representations of different dimensions. After flattening, these representations are concatenated along the channel dimension to form a unified high-dimensional feature vector. This fused feature vector is then passed to a fully connected layer, and the final class probability distribution is obtained through the Softmax activation function. The calculation formula for the Softmax function is as follows:

(7) $${\hat{y}}_{i}=\frac{e^{z_{i}}}{\sum_{j=1}^{C}e^{z_{j}}},\;i=1,2,\ldots ,C,$$

where $z_{i}$ represents the response of the *i*-th neuron in the output layer, *C* denotes the total number of classes, and ${\hat{y}}_{i}$ is the predicted probability for the *i*-th class.

Depending on the task configuration of each dataset, the number of neurons in the output layer is adjusted accordingly. For the EEGMMIDB dataset, the output layer consists of 5 neurons, corresponding to the five task classes: eyes-closed resting, left hand MI, right hand MI, both hands MI, and feet MI. For the BCIIV2A dataset, the output layer contains 4 neurons, representing the four task classes: left hand, right hand, feet, and tongue MI. This output structure ensures that MSSTNet can flexibly adapt to the classification requirements of different datasets, while maintaining stable and accurate decoding performance across multi-source data fusion conditions.

## 3. Experiments and Results

### 3.1. Training Procedure

The experiments in this study were conducted on a Windows 11 Professional Workstation, equipped with a 13th Gen Intel Core i9 processor, an NVIDIA GeForce RTX 4060 GPU, and 64 GB of DDR5 memory. The deep learning framework was implemented using PyTorch 2.4.1, while EEG data preprocessing was performed using the MNE 1.8.0 package.

To ensure the stability of the training process and generalization of the evaluation results, the data were split into training, validation, and test sets with a ratio of 7:1:2. Prior to formal training, the data were split into training, validation, and test sets to prevent information leakage. Subsequently, sliding window-based sample augmentation was applied to both sets.

For model training, data from each subject were used independently for training and evaluation, following a subject-dependent modeling approach. The Adam optimizer was employed with an initial learning rate of 0.01, and dynamic learning rate adjustments were made using the ReduceLROnPlateau strategy [30]. CrossEntropyLoss was used as the loss function, and the maximum number of training epochs was set to 80. After each epoch, the model was evaluated on the validation set, and the best model parameters were dynamically saved based on the validation accuracy.

### 3.2. Evaluation Methods

To comprehensively evaluate the decoding performance of MSSTNet on multi-class MI tasks, this study employs three commonly used evaluation metrics: confusion matrix, accuracy, and kappa coefficient. Additionally, gradient-weighted class activation mapping (Grad-CAM) is utilized to enhance the interpretability of MSSTNet by visualizing the spatial and spectral features that are most relevant for classification.

(1) Confusion Matrix

The confusion matrix provides a clear visualization of the model’s classification accuracy and the extent of misclassification across different tasks [31]. For an *N*-class classification task, the confusion matrix is an N×N matrix, where the element in the *i*-th row and *j*-th column represents the number of samples that actually belong to class *i* but are predicted as class *j*. The elements along the main diagonal represent the number of correctly classified samples. The closer the data points are to the main diagonal, the stronger the model’s discriminative ability.

(2) Accuracy

Accuracy is the fundamental metric for evaluating the overall classification performance of a model, defined as follows:

(8) $$Accuracy=\frac{TP+TN}{TP+TN+FP+FN},$$

where TP represents the number of positive samples correctly classified, TN represents the number of negative samples correctly classified, FP represents the number of negative samples incorrectly classified as positive, and FN represents the number of positive samples incorrectly classified as negative. For multi-class tasks, this metric can be simplified as

(9) $$Accuracy=\frac{\sum_{i=1}^{N}C_{ii}}{\sum_{i=1}^{N}\sum_{j=1}^{N}C_{ij}},$$

where $C_{ij}$ are the elements of the confusion matrix, $C_{ii}$ represents the main diagonal elements (i.e., the correctly classified samples), and *N* represents the number of classification categories.

(3) Kappa Coefficient

The kappa coefficient is a classification consistency metric that accounts for random agreement [32], providing a more accurate reflection of the model’s discriminative ability compared to accuracy. It is defined as follows:

(10) $$k=\frac{p_{o}-p_{e}}{1-p_{e}},$$

where $p_{o}$ represents the observed agreement (i.e., classification accuracy), and $p_{e}$ corresponds to the expected agreement under random classification assumptions, calculated from the marginal distributions of the predicted and actual class labels [33].

The kappa coefficient ranges from [−1,1]: A value of k=1 indicates perfect agreement, meaning the model’s predictions match the true labels exactly, reflecting high discriminative ability and stability. A value of k=0 indicates that the model’s performance is no better than random classification, suggesting the model lacks effective classification ability. A value of k<0 indicates a negative correlation between the predicted and true labels, suggesting systematic misclassification by the model.

(4) Grad-CAM Visualization

Grad-CAM is a visualization technique that helps interpret the decisions of convolutional neural networks by producing class-specific heatmaps [34]. Grad-CAM uses the gradients of the target class with respect to the feature maps in the last convolutional layer to generate a class activation map, highlighting which regions of the input are most influential for the model’s decision.

In order to obtain Grad-CAM, the gradients of the target class’s score with respect to the feature maps from the final convolutional layer are first calculated. These gradients are then globally averaged and used to weight the feature maps. The weighted feature maps are summed, and the resulting values are passed through a ReLU activation function. This produces the final class activation map, which is overlaid on the input signal to visually highlight the most influential regions for the model’s prediction.

The intensity of the Grad-CAM heatmap reflects the degree to which each region influences the model’s classification decision. Regions with higher activation values correspond to stronger model attention and greater contribution to the final decision.

### 3.3. Experimental Results

This section presents the experimental results in three parts: first, the classification performance of the model across different subjects is demonstrated; second, an ablation study is conducted to analyze the contribution of each feature extraction branch; third, a performance comparison is made with existing methods; and finally, Grad-CAM visualizations are presented to analyze the model’s decision-making process and enhance its neurophysiological interpretability.

#### 3.3.1. Confusion Matrix

**Figure 6:**
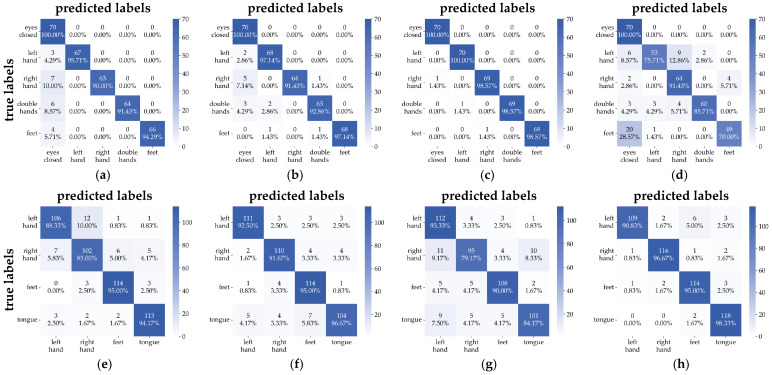
Confusion matrices of MSSTNet on representative subjects from the two datasets. (**a**–**d**) Subjects S001, S004, S007, and S010 from the EEGMMIDB dataset. (**e**–**h**) Subjects A02, A04, A06, and A08 from the BCIIV2A dataset.

To provide a comprehensive evaluation of the model’s classification performance across different subjects, Figure 6 presents the confusion matrix results for several subjects from both datasets. Specifically, Figure 6a–d correspond to subjects S001, S004, S007, and S010 from the EEGMMIDB dataset, while Figure 6e–h correspond to subjects A02, A04, A06, and A08 from the BCIIV2A dataset.

On the EEGMMIDB dataset, MSSTNet demonstrates strong discriminative ability for the five-class task, with most samples being correctly classified. Particularly in the eyes-closed category, the model achieves high recognition accuracy. This is attributed to the fact that during the eyes-closed resting state, the brain is in a relaxed non-task state, and the EEG signals exhibit activity patterns that are distinct from those observed during MI tasks [35]. These stable brainwave patterns are easier for the model to recognize and differentiate. On the BCIIV2A dataset, the predictions for each category are predominantly concentrated along the main diagonal, reflecting the high accuracy of the model.

These results visually demonstrate MSSTNet’s stability and robustness across different tasks and subjects, validating its effectiveness in multi-class MI-EEG decoding.

#### 3.3.2. Ablation Study

To systematically assess the contribution of each feature extraction branch within the overall framework, multiple architectural variants of MSSTNet were evaluated through an ablation study. MSSTNet consists of four parallel branches: Branch 1 extracts spatial features, Branch 2 captures spectral features, Branch 3 extracts spatial-spectral joint features, and Branch 4 captures temporal features. Based on this structure, the following model variants were constructed:

(1) Single-branch models: Variants using only one of Branches 1, 2, 3, or 4 were evaluated to assess the independent modeling capabilities of each feature dimension.
(2) Two-branch combination models: Four dual-branch variants (Branch 1+2, Branch 1+4, Branch 2+4, and Branch 3+4) were developed to explore the collaborative modeling effects of different feature dimensions and to analyze the complementarity among spatial, spectral, and temporal information.
(3) Multi-branch combination models: In addition to the full MSSTNet architecture, variants combining Branch 1+2+3 and Branch 1+2+4 were also evaluated to assess the comprehensive performance under multi-dimensional feature fusion.

All variants were independently trained using a consistent training strategy and evaluated on a fixed data partitioning scheme for each subject. The average classification accuracy and kappa coefficient on the test set were computed for each variant, and the results are summarized in Table 1 and Table 2.

The results of the single-branch models, as shown in Table 1 and Table 2, reveal several key findings. Among the four branches, the spectral branch shows the lowest performance, suggesting that while the spectral dimension captures EEG energy distribution, its discriminative ability is limited without spatial or temporal context. The spatial branch performs slightly better, indicating that spatial structural information–derived from electrode placement and cortical activation–provides better class separability when modeled independently. The spatial-spectral joint branch improves performance further, demonstrating that combining spatial and spectral features allows for more complex neural activity patterns and cross-band or channel interactions, enhancing MI classification. The temporal branch performs slightly below the spatial-spectral branch across both datasets but consistently outperforms both the spatial and spectral branches. This indicates that temporal modeling based on 2D time-domain EEG preserves the dynamic evolution of neural activity and provides strong discriminative power. Notably, the temporal branch still performs competitively without spectral-domain processing, underscoring the effectiveness of end-to-end temporal modeling for MI-EEG tasks.

Next, the results of the dual-branch models, as shown in Table 1 and Table 2, are examined. Combining the temporal branch with any other branch shows significant performance improvements. Specifically, the combination of the temporal and spatial-spectral branches yields the best results. This indicates strong complementarity between temporal features and spatial-spectral representations, enabling effective modeling of coordinated variations in spectral dynamics and spatial distributions associated with MI tasks. In contrast, the combination of the spectral and spatial branches, although outperforming each branch independently, shows more limited performance gains. This suggests that their collaborative capacity is constrained without temporal modeling.

Finally, the results of the multi-branch models in Table 1 and Table 2 show that as the number of integrated branches increases, the overall model performance consistently improves. Among the three-branch configurations, the combination of spatial, spectral, and spatial-spectral branches incorporates all PSD-based features. However, without temporal modeling, its performance is still noticeably lower than models that include the temporal branch. The integration of spatial, spectral, and temporal branches results in the highest performance, demonstrating that temporal modeling complements spatial and spectral features by enhancing both the completeness and discriminative power of the feature representations.

Ultimately, MSSTNet combines all four branches, capturing spatial, spectral, spatial-spectral joint, and temporal features. It achieves the highest average classification accuracy and kappa coefficient across both datasets, clearly demonstrating the significant advantages of multi-dimensional feature fusion in decoding complex neural activity patterns.

#### 3.3.3. Performance Comparison

To comprehensively evaluate the effectiveness of the proposed MSSTNet in MI-EEG decoding tasks, its performance is compared with several representative models: the lightweight end-to-end network EEGNet [15]; the traditional shallow network ShallowFBCSPNet based on FBCSP features [14]; the multi-scale architecture-driven EEGInception [36]; the graph neural network-based CRGNet [37]; and the recently proposed spatial-spectral joint modeling method BrainGridNet [19]. These methods have demonstrated strong performance in MI-EEG decoding and represent mainstream paradigms such as spectral modeling, spatial modeling, graph-structured modeling, and multi-scale feature extraction. They thus provide a comprehensive and diverse foundation for benchmarking and validating MSSTNet’s performance from multiple perspectives.

As shown in Table 3 and Table 4, MSSTNet consistently achieves the highest average classification accuracy and kappa coefficient among all compared methods on both the EEGMMIDB and BCIIV2A datasets. On the EEGMMIDB dataset, MSSTNet achieves an average accuracy of 86.34% and a kappa coefficient of 0.829, significantly outperforming methods such as BrainGridNet. On the more challenging BCIIV2A dataset, MSSTNet also demonstrates strong decoding capability, achieving an average accuracy of 83.43% and a kappa coefficient of 0.779, clearly surpassing other mainstream approaches. These results further validate the effectiveness of the multi-branch fusion strategy in extracting discriminative features across multiple dimensions, emphasizing the synergistic advantage of integrating temporal modeling with spectral-spatial feature fusion. This provides new insights into improving the accuracy of MI-EEG decoding.

#### 3.3.4. Grad-CAM Visualization Analysis

Despite experimental results demonstrating superior classification performance in both ablation and comparative experiments, the internal decision-making mechanisms of the model remain opaque and lack intuitive interpretability. To improve the neurophysiological interpretability of the model and gain deeper insights into its decision-making process in MI-EEG decoding tasks, this study applies Grad-CAM to visualize the contributions of each feature extraction branch.

Since each branch of the model is responsible for extracting distinct types of features, this section presents a visualization analysis of the first three feature branches: the spatial, spectral, and spatial-spectral joint branches. The aim is to examine the regions each branch focuses on across different task categories. For clarity and representativeness, subject S007 from the EEGMMIDB dataset and subject A03 from the BCIIV2A dataset were selected for illustration. Additionally, visualization analyses conducted on other subjects reveal consistent activation patterns across task categories within each branch, further confirming the robustness and generalizability of the model’s feature extraction capabilities.

(1) Branch 1: Spatial Feature Extraction Branch

**Figure 7:**
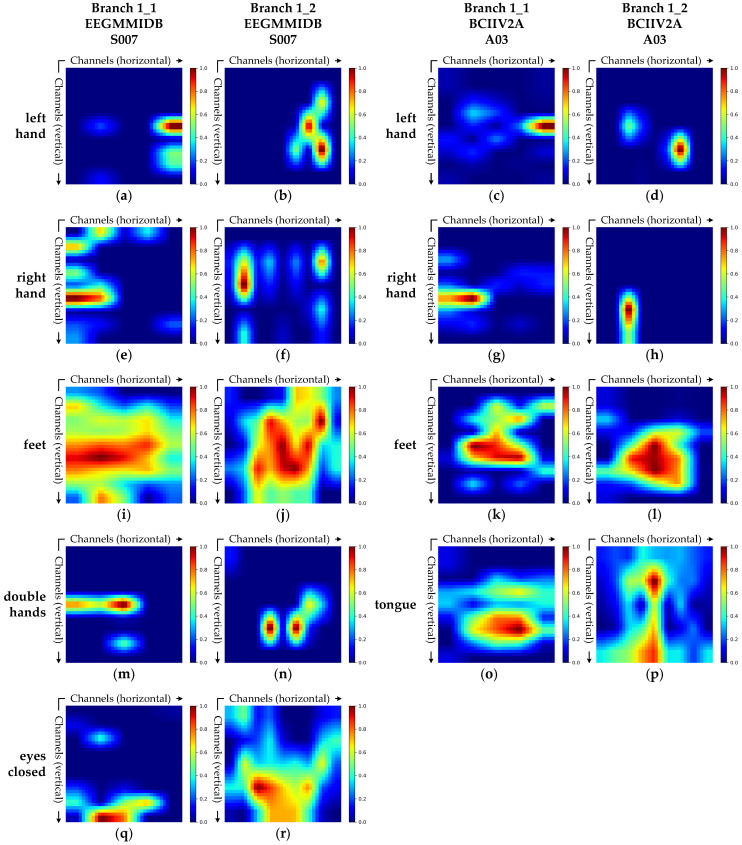
Grad-CAM visualizations of the spatial feature extraction branch (Branch 1) for subject S007 from the EEGMMIDB dataset and subject A03 from the BCIIV2A dataset. (**a**–**d**) Grad-CAM heatmaps for the left hand task in Branch 1_1 and Branch 1_2 for subject S007 and subject A03. (**e**–**h**) The right hand task for subject S007 and subject A03. (**i**–**l**) The feet task for subject S007 and subject A03. (**m**,**n**) The double hands task for subject S007. (**o**,**p**) The tongue task for subject A03. (**q**,**r**) The eyes-closed task for subject S007.

Figure 7 presents the Grad-CAM visualizations of the spatial feature extraction branch in MSSTNet for two representative subjects, highlighting the spatial electrode regions that the branch attends to under different tasks.

This branch consists of two parallel submodules, Branch 1_1 and Branch 1_2. Each submodule uses convolutional kernels of size 1×n and n×1, respectively, to extract spatial features along the vertical and horizontal electrode dimensions. In Branch 1_1, the second depthwise convolution is applied without padding in the horizontal direction, resulting in lateral compression of the feature maps. During Grad-CAM visualization, these compressed feature maps are upsampled, which introduces horizontal stretching in the resulting heatmaps. Consequently, the visualizations primarily reflect the model’s attention along the vertical electrode axis, making the vertical activation regions more prominent. In contrast, Branch 1_2 applies its second depthwise convolution without padding in the vertical direction, so its Grad-CAM heatmaps highlight regions along the horizontal axis. Together, these two submodules provide complementary insights into the model’s spatial attention, revealing how MSSTNet captures discriminative spatial patterns and shows sensitivity to the spatial distribution of neurophysiological signals.

Taking the left hand MI task as an example, the Grad-CAM results in Figure 7a,c reveal that Branch 1_1 predominantly focuses on the central region along the vertical channel dimension, with consistent patterns across subjects from both datasets. Further examination of Figure 7b,d demonstrates that Branch 1_2 exhibits strong activation in the right hemisphere along the horizontal channel dimension, with activation concentrated in channels corresponding to the right hemisphere. These observations indicate that Branch 1 focuses on the central-right spatial region during the left hand MI task, aligning with the well-established neurophysiological principle that contralateral motor cortices are activated during unilateral limb movement [38]. This confirms the neurophysiological interpretability of the spatial features extracted by this branch.

Beyond the left hand task, Branch 1 also exhibits distinct and physiologically meaningful spatial activation patterns for other tasks. As shown in Figure 7e–h, the branch primarily focuses on the central-left area during the right hand MI task, consistent with contralateral cortical activation associated with right hand movement [38]. In the feet task, as illustrated in Figure 7i–l, attention is directed to central midline scalp regions, consistent with the somatotopic organization of the foot area in the medial sensorimotor cortex [39]. For the both hands task, as shown in Figure 7m,n, Branch 1 shows strong bilateral activation, reflecting the cooperative cortical response of bimanual motor execution [40]. For the tongue MI task, as illustrated in Figure 7o,p, the model focuses on lower and lateral brain regions associated with orofacial movement, consistent with the cortical localization of tongue activity in the inferior sensorimotor area [41]. Lastly, for the eyes-closed task, as shown in Figure 7q,r, activation is concentrated in the posterior brain region, particularly around the O1 and O2 channels, reflecting increased activity in the visual cortex during eye closure [42].

In summary, the spatial feature extraction branch of MSSTNet consistently demonstrates attention patterns that align with established neurophysiological principles across various MI tasks. It accurately focuses on brain regions associated with specific motor functions, showing a strong ability to discriminate spatial electrode structures. This not only confirms the effectiveness of the model in extracting spatial features but also reinforces the interpretability of the MSSTNet architecture. Such neurophysiologically meaningful spatial representations provide a solid foundation for reliable decoding of MI-EEG signals.

(2) Branch 2: Spectral Feature Extraction Branch

**Figure 8:**
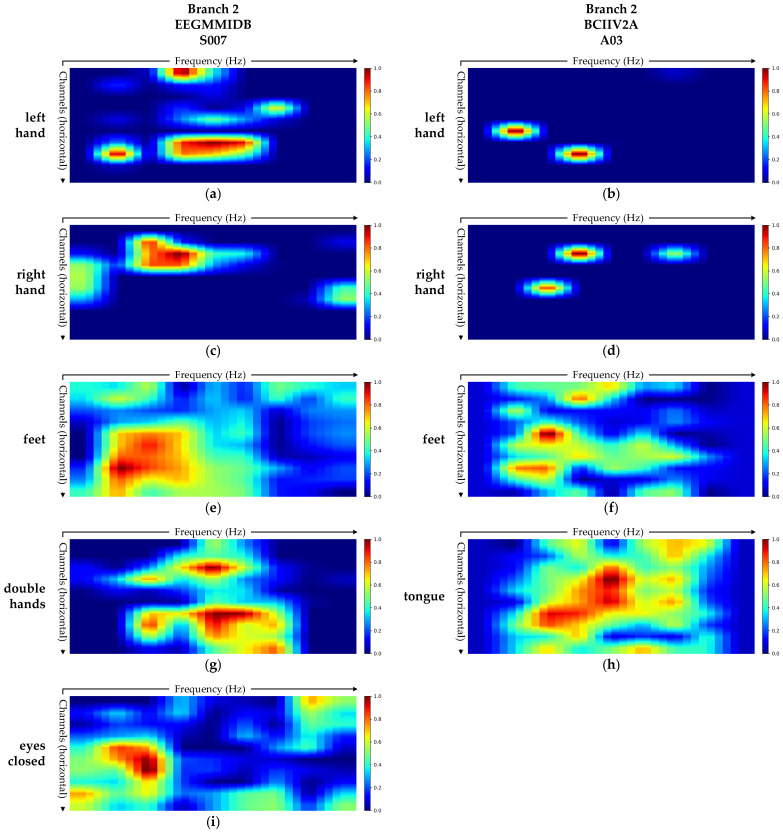
Grad-CAM visualizations of the spectral feature extraction branch (Branch 2) for subject S007 from the EEGMMIDB dataset and subject A03 from the BCIIV2A dataset. (**a**,**b**) Grad-CAM heatmaps for the left hand task in Branch 2 for subject S007 and subject A03. (**c**,**d**) The right hand task for subject S007 and subject A03. (**e**,**f**) The feet task for subject S007 and subject A03. (**g**) The double hands task for subject S007. (**h**) The tongue task for subject A03. (**i**) The eyes-closed task for subject S007.

Figure 8 presents the Grad-CAM visualizations of the spectral feature extraction branch of MSSTNet for two representative subjects, revealing the frequency-region attention patterns associated with different tasks.

Taking the left hand MI task as an example, the visualizations in Figure 8a,b show that Branch 2 exhibits strong activation in both low-frequency and mid-frequency bands across both subjects [43]. These frequency ranges correspond to the typical α (8–13 Hz) and β (13–30 Hz) rhythms, which are known to be primarily involved in motor-related cortical rhythm suppression. Beyond the left hand class, Branch 2 also demonstrates physiologically plausible frequency attention patterns across other tasks. As shown in Figure 8a–h, the most prominent activation regions across all MI tasks are predominantly within the α and β rhythms [40,42,43]. In the feet MI task, illustrated in Figure 8e,f, the frequency attention is more concentrated in lower frequency range compared to the left hand and right hand tasks. This suggests that Branch 2 places greater emphasis on low-frequency neural activity in this task. Furthermore, as shown in Figure 8i, activation during the eyes-closed task is primarily concentrated within the α rhythm band, which aligns with the neurophysiological mechanism of enhanced α activity in the visual cortex during eye closure [44].

In summary, Branch 2 effectively captures key frequency-band variations associated with motor-related tasks across different MI categories. The extracted spectral attention patterns closely align with well-established neural rhythmic characteristics. This validates the model’s ability in frequency-domain modeling and demonstrating the neurophysiological interpretability of this branch in MI decoding.

(3) Branch 3: Spatial-Spectral Joint Feature Extraction Branch

**Figure 9:**
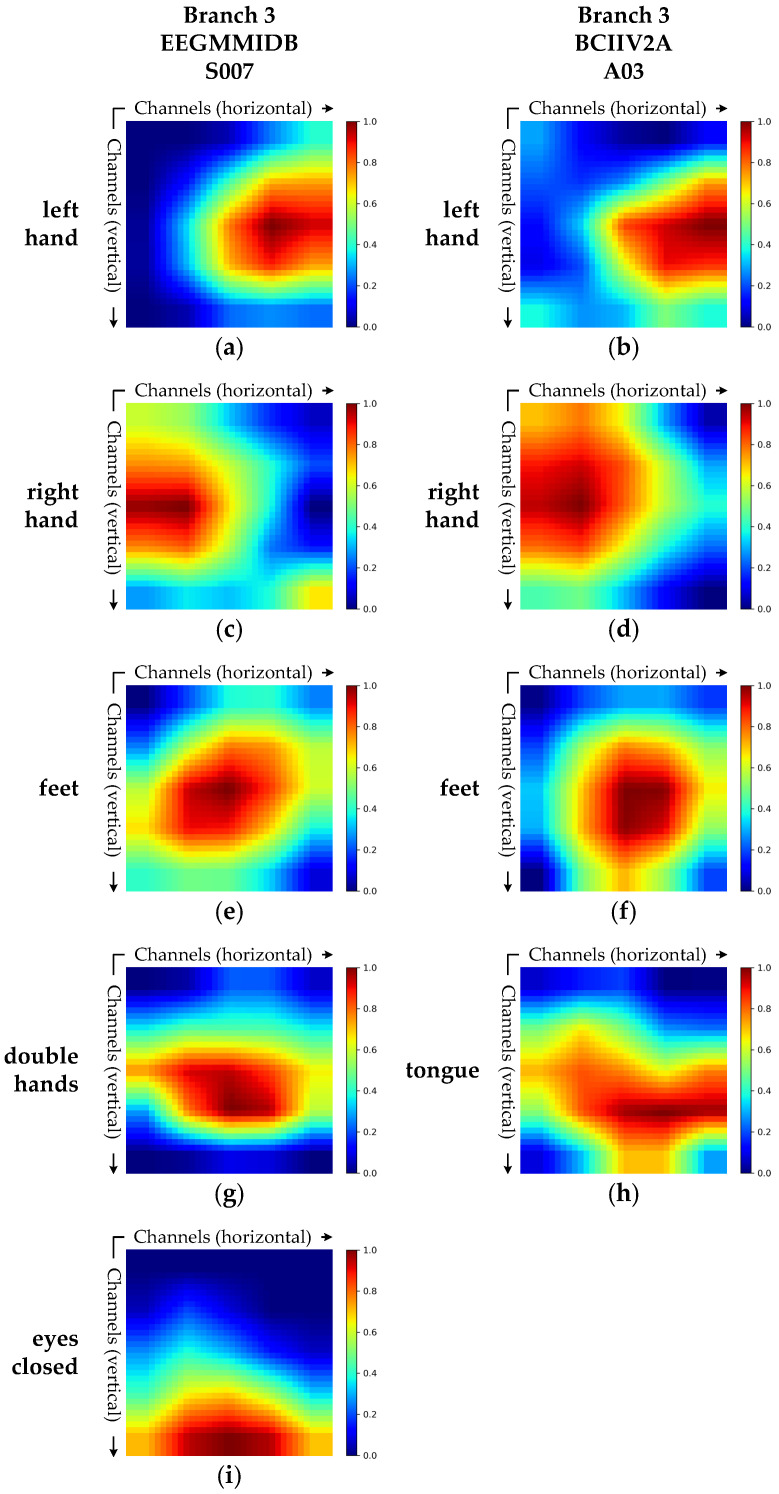
Grad-CAM visualizations of the spatial-spectral joint extraction branch (Branch 3) for subject S007 from the EEGMMIDB dataset and subject A03 from the BCIIV2A dataset. (**a**,**b**) Grad-CAM heatmaps for the left hand task in Branch 3 for subject S007 and subject A03. (**c**,**d**) The right hand task for subject S007 and subject A03. (**e**,**f**) The feet task for subject S007 and subject A03. (**g**) The double hands task for subject S007. (**h**) The tongue task for subject A03. (**i**) The eyes-closed task for subject S007.

Figure 9 presents the Grad-CAM visualizations of the spatial-spectral joint feature extraction branch for two representative subjects. These visualizations highlight the key spatial channel regions that the branch attends to when decoding different tasks.

Branch 3 utilizes 3D convolution modules to jointly model spatial and spectral dimensions, allowing the integration of spectral context while extracting spatial features. Consequently, its activation regions exhibit both spatially localized patterns and spectrally discriminative characteristics. While the Grad-CAM visualizations primarily emphasize spatial responses along the channel dimension, these responses are derived from feature maps generated through joint spectral-spatial modeling. As a result, they more accurately reflect the model’s attention to complex neural patterns.

As shown in Figure 9a,b, Branch 3 exhibits strong activation in the central region of the right hemisphere during the left hand MI task. In Figure 9c,d, the activation shifts to the central region of the left hemisphere for the right hand MI task. For the feet task, illustrated in Figure 9e,f, the activation is concentrated in the centro-parietal area, consistent with the anatomical location of the foot motor cortex [39]. As shown in Figure 9g, the both hands task elicits widespread responses across both hemispheres. Figure 9h reveals broad activation across the lower and lateral brain regions during the tongue MI task. Lastly, Figure 9i demonstrates that the eyes-closed task triggers activation in the posterior brain region, aligning with the known synchronization enhancement in the visual cortex [42].

Overall, Branch 3 displays spatial activation patterns that align closely with established neurophysiological principles across different tasks. These patterns are consistent with those observed in Branch 1 visualizations, further validating the model’s spatial feature extraction mechanism. Compared to Branches 1 and 2, the activation regions in Branch 3 are more compact and focused, highlighting its enhanced ability to extract discriminative features through integrated spatial-spectral modeling.

## 4. Discussion

To address the limitations of current MI-EEG decoding methods in jointly modeling spatial, spectral, and temporal features, this study introduces a multi-branch deep neural network, termed MSSTNet. The network accepts both a 3D PSD representation and 2D time-domain EEG signals as dual inputs and is equipped with four dedicated feature extraction branches designed to capture spatial, spectral, spatial-spectral joint, and temporal features, respectively. Lightweight DSC modules are incorporated to significantly reduce the computational complexity without compromising the representational capacity. Experimental evaluations on two publicly available datasets, EEGMMIDB and BCIIV2A, demonstrate that MSSTNet significantly outperforms state-of-the-art approaches in both the classification accuracy and kappa coefficient, validating its superiority in multi-dimensional feature modeling. Additionally, ablation experiments confirm the independent contributions and complementarity of each branch, while Grad-CAM-based visualizations elucidate the critical spatial and spectral patterns prioritized by the model across different tasks. These patterns align well with established neurophysiological principles, providing strong physiological interpretability for the model’s decision-making process.

Among these findings, the ablation experiments show that the spatial-spectral joint branch, even when used independently, outperforms the dual-branch combination of the spatial and spectral branches. This advantage is attributed to its explicit cross-channel and cross-frequency modeling, enabled by the 3D DSC modules during feature extraction. This allows the convolutional layers to capture richer cross-dimensional interaction patterns. In contrast, the spatial-only and spectral-only branches perform independent modeling within their respective dimensions. They rely on simple feature concatenation at higher layers, lacking early-stage cross-dimensional fusion. This limitation restricts their overall discriminative capacity. These results suggest that hierarchical convolution-based spatial-spectral coupling provides substantial benefits for improving MI decoding performance, compared to late-stage feature concatenation alone.

However, although the spatial-spectral joint branch demonstrates superior performance when used independently, its combination with the temporal branch results in lower performance than the combination of the spatial and spectral branches. This phenomenon may be attributed to differences in feature diversity and complementarity. The spatial and spectral branches perform independent modeling within their respective dimensions, preserving more low-level and non-overlapping feature patterns. These patterns form stronger complementary effects when integrated with the temporal branch’s dynamic features. In contrast, the spatial-spectral joint branch completes cross-channel and cross-frequency coupling during early feature extraction. This results in high-level representations that partially overlap with the temporal branch’s time-related patterns, limiting the incremental contribution of new discriminative information. These findings suggest that, in multi-branch fusion strategies, maintaining relative independence and orthogonality across feature dimensions may be more advantageous than premature multi-dimensional coupling, as it enhances the complementary role of temporal features.

To further elucidate the significance of this study, the performance of MSSTNet was compared with findings reported in the relevant literature. The results indicate that MSSTNet’s superiority in multi-dimensional feature modeling is consistent with the previous studies. It is this consistency that highlights the benefits of multi-feature fusion for enhancing MI-EEG decoding performance. For example, prior research has demonstrated that the α and β rhythms exhibit prominent event-related desynchronization characteristics during MI tasks [38,40]. In this study, both the spectral branch and the spatial-spectral joint branch effectively captured strong activations within these frequency bands. Moreover, the spatial attention patterns revealed by model visualizations closely align with classical neurophysiological theories: contralateral motor cortex activation for left hand and right hand tasks [38]; central parietal activation for both-feet tasks [39]; inferior motor cortex activation for tongue tasks [41]; and enhanced occipital α rhythms during eyes-closed tasks [44]. This high degree of agreement with established physiological principles not only validates the model’s ability to capture multi-dimensional features but also underscores its neurophysiological interpretability.

In summary, the main contributions of this study to the field of MI-EEG decoding are as follows:

(1) This study proposes MSSTNet, a multi-branch deep neural network that takes both 3D PSD representations and 2D time-domain EEG signals as dual inputs. These inputs enable the model to capture spatial, spectral, and temporal features effectively.
(2) MSSTNet incorporates four specialized feature extraction branches and employs DSC modules as core components. This design achieves a balance between representational capacity and computational efficiency, ensuring robust decoding performance while reducing the computational overhead.
(3) On the EEGMMIDB and BCIIV2A datasets, MSSTNet achieves accuracies of 86.34% and 83.43%, and kappa coefficients of 0.829 and 0.779, respectively, outperforming representative methods including EEGNet, ShallowFBCSPNet, EEGInception, CRGNet, and BrainGridNet.
(4) Ablation experiments systematically quantify the independent contributions and complementarity of each branch. They reveal that hierarchical convolution-based cross-dimensional modeling more effectively captures spatial-spectral interactions during feature extraction. Meanwhile, the temporal branch provides essential complementary information, further enhancing overall discriminative performance.
(5) Grad-CAM visualizations show that the model’s attention patterns align closely with established neurophysiological principles, thereby corroborating the physiological plausibility of its decision-making process from an interpretability perspective.

Despite the promising performance of MSSTNet in multi-dimensional feature modeling and interpretability, several limitations and challenges remain:

(1) Although the multi-branch architecture effectively integrates spatial, spectral, and temporal features, the current fusion strategy relies on simple concatenation. This approach lacks explicit cross-dimensional interaction modeling, which may constrain further improvements in fusion effectiveness.
(2) The training and evaluation in this study were conducted exclusively in a subject-dependent setting. The model’s generalization ability under cross-subject transfer or few-shot scenarios, which is critical for practical BCI system applications, has yet to be validated.
(3) While the DSC modules outperform 2D standard convolutions in reducing parameter count and computational overhead, the overall network depth of MSSTNet remains relatively large. So its deployment efficiency on low-power or embedded devices could still be optimized compared with certain lightweight architectures, such as BrainGridNet.

Therefore, while maintaining discriminative performance, future research could explore the incorporation of cross-branch attention mechanisms [45] to enable adaptive weighting of features from different dimensions. Additionally, data alignment techniques, such as Euclidean alignment [46], could be employed prior to model training to reduce inter-subject distributional discrepancies. This could be combined with transfer learning or few-shot strategies to reduce individual calibration costs and enhance the stability of cross-subject performance. Furthermore, further investigation into structural compression and operator optimization could facilitate the development of more efficient and lightweight network architectures. This would promote the real-time application of MI-EEG decoding in neurorehabilitation training, providing more precise and personalized rehabilitation support for patients with motor impairments, such as stroke.

## 5. Conclusions

This study focuses on the core objective of comprehensively capturing multi-dimensional EEG features, proposing MSSTNet to efficiently decode MI-EEG signals. MSSTNet is a multi-branch deep neural network that integrates spatial, spectral, spatial-spectral joint, and temporal features for comprehensive modeling. By constructing a three-dimensional power spectral density (3D PSD) tensor in conjunction with two-dimensional time-domain EEG signals, the model preserves both spatial topological structure and spectral distribution information. This approach supplements the ability to capture temporal dynamics, thereby substantially enhancing the completeness of feature representation. All four parallel feature extraction branches employ depthwise separable convolution (DSC) modules, enabling effective fusion of complementary multi-dimensional information while maintaining low computational overhead, thus ensuring robust classification performance. In multi-class MI tasks on two publicly available datasets, EEGMMIDB and BCIIV2A, MSSTNet achieved significantly higher average classification accuracy and kappa coefficients compared to several state-of-the-art methods, demonstrating its superiority across different subjects and task conditions. Visualizations based on gradient-weighted class activation mapping (Grad-CAM) identified key brain regions and frequency bands prioritized during the decision-making process. These visualizations reveal strong consistency with established neurophysiological mechanisms, thereby enhancing the model’s credibility and neurophysiological interpretability. Overall, MSSTNet not only achieves comprehensive modeling of spatial, spectral, and temporal dynamic features but also delivers notable advances in both classification performance and interpretability, fully addressing the initial research objectives. Future work will focus on further optimizing multi-feature fusion strategies and exploring the potential of the model in cross-subject transfer learning and real-time online BCI applications.

## Figures and Tables

**Table 1:** Comparison of average decoding accuracy (%) and kappa coefficient across MSSTNet variants on the EEGMMIDB dataset.

Model	Subjects										Avg. Acc	Avg. Kappa
	S001	S002	S003	S004	S005	S006	S007	S008	S009	S010		
Branch 1	85.14	80.86	78.29	87.71	73.14	69.43	88.57	74.57	70.00	76.57	78.43	0.729
Branch 2	83.14	80.00	77.71	87.43	73.14	69.14	88.00	73.14	68.86	74.86	77.54	0.718
Branch 3	88.29	83.43	80.86	91.14	75.14	72.29	92.00	75.43	72.29	78.57	80.94	0.761
Branch 4	86.29	81.43	79.71	89.14	74.29	70.57	90.57	75.43	70.29	76.86	79.46	0.742
Branch 1+2	87.14	82.86	81.14	90.57	74.86	71.71	91.43	75.71	71.14	77.43	80.40	0.754
Branch 1+4	87.71	83.71	82.57	90.29	76.00	71.14	93.14	77.14	71.71	77.71	81.11	0.763
Branch 2+4	88.29	82.86	81.71	90.86	75.43	71.43	92.29	75.71	72.57	78.57	80.97	0.762
Branch 3+4	90.00	84.00	82.86	92.57	77.14	72.57	94.29	77.43	73.71	79.71	82.43	0.780
Branch 1+2+3	91.43	84.57	83.43	92.86	78.00	74.00	96.29	78.00	75.14	81.43	83.52	0.799
Branch 1+2+4	93.14	86.00	85.43	94.57	79.71	75.71	98.00	80.00	77.14	83.71	85.43	0.818
**MSSTNet**	**94.29**	**87.43**	**87.14**	**95.71**	**80.29**	**76.00**	**99.14**	**81.43**	**77.43**	**84.57**	**86.34**	**0.829**

*Notes.* Avg. acc denotes average accuracy; Avg. kappa denotes average kappa coefficient; Bold values highlight the best performance among all model variants for each subject and overall.

**Table 2:** Comparison of average decoding accuracy (%) and kappa coefficient across MSSTNet variants on the BCIIV2A dataset.

Model	Subject									Avg. Acc	Avg. Kappa
	A01	A02	A03	A04	A05	A06	A07	A08	A09		
Branch 1	83.54	60.21	89.17	68.33	51.46	67.08	85.83	83.96	81.67	74.58	0.661
Branch 2	83.75	61.04	81.25	70.00	52.08	65.00	82.29	77.50	78.12	72.34	0.631
Branch 3	89.17	67.50	86.04	73.96	58.33	64.58	82.71	87.82	82.29	76.93	0.693
Branch 4	91.04	60.42	85.21	76.67	56.25	59.79	81.46	87.50	82.08	75.60	0.675
Branch 1+2	90.00	62.08	87.29	63.96	62.71	70.83	88.54	84.58	76.25	76.25	0.683
Branch 1+4	89.58	66.25	92.71	70.42	63.54	71.67	87.29	80.83	78.96	77.92	0.706
Branch 2+4	83.54	67.92	86.88	78.33	61.25	58.33	87.08	87.71	83.96	77.22	0.696
Branch 3+4	89.79	67.71	88.33	67.50	62.29	72.71	88.75	87.29	84.58	78.77	0.717
Branch 1+2+3	88.54	65.00	92.50	73.75	63.54	72.92	91.04	87.29	86.46	80.12	0.736
Branch 1+2+4	91.67	68.95	94.17	76.67	65.62	73.54	91.87	87.92	82.34	82.01	0.765
**MSSTNet**	**92.29**	**70.83**	**95.83**	**77.29**	**67.92**	**75.21**	**92.92**	**88.75**	**89.79**	**83.43**	**0.779**

*Notes.* Avg. acc denotes average accuracy; Avg. kappa denotes average kappa coefficient; Bold values highlight the best performance among all model variants for each subject and overall.

**Table 3:** Comparison of average decoding accuracy (%) and kappa coefficient between MSSTNet and existing methods on the EEGMMIDB dataset.

Model	Subjects										Avg. Acc	Avg. Kappa
	S001	S002	S003	S004	S005	S006	S007	S008	S009	S010		
EEGNet	68.57	65.14	69.71	59.43	56.86	54.86	78.86	63.43	53.71	61.71	63.40	0.537
ShallowFBCSPNet	79.71	87.43	77.14	67.43	58.29	61.43	89.43	74.29	60.29	71.14	72.83	0.682
EEGInception	77.71	76.00	78.86	72.86	69.43	73.14	80.29	73.71	65.14	64.29	74.13	0.700
CRGNet	82.28	**89.43**	74.57	73.43	68.29	63.71	94.57	80.86	58.29	68.57	76.16	0.712
BrainGridNet	89.71	83.14	75.71	87.71	75.43	**78.86**	93.43	77.43	75.71	74.29	81.90	0.756
**MSSTNet**	**94.29**	87.43	**87.14**	**95.71**	**80.29**	76.00	**99.14**	**81.43**	**77.43**	**84.57**	**86.34**	**0.829**

*Notes.* Avg. acc denotes average accuracy; Avg. kappa denotes average kappa coefficient; Bold values highlight the best performance among all compared methods for each subject and overall.

**Table 4:** Comparison of average decoding accuracy (%) and kappa coefficient between MSSTNet and existing methods on the BCIIV2A dataset.

Model	Subjects									Avg. Acc	Avg. Kappa
	A01	A02	A03	A04	A05	A06	A07	A08	A09		
EEGNet	83.43	59.43	85.43	66.28	57.14	53.71	86.86	81.14	84.57	73.11	0.662
ShallowFBCSPNet	86.67	62.29	89.79	72.09	56.46	57.92	90.83	80.42	78.96	75.05	0.689
EEGInception	85.43	65.58	94.28	71.14	55.71	68.57	88.00	85.43	87.71	77.98	0.752
CRGNet	81.67	68.54	88.33	72.92	62.29	70.42	**93.13**	86.86	76.46	77.85	0.748
BrainGridNet	90.83	63.54	90.42	**80.63**	**77.50**	71.25	86.25	77.71	83.33	80.16	0.760
**MSSTNet**	**92.29**	**70.83**	**95.83**	77.29	67.92	**75.21**	92.92	**88.75**	**89.79**	**83.43**	**0.779**

*Notes.* Avg. acc denotes average accuracy; Avg. kappa denotes average kappa coefficient; Bold values highlight the best performance among all compared methods for each subject and overall.

## Data Availability

Publicly available datasets were used in this study. These data can be found here: https://www.bbci.de/competition/download/competition_iv/BCICIV_2a_gdf.zip (accessed on 25 July 2025); https://www.physionet.org/content/eegmmidb/get-zip/1.0.0/ (accessed on 25 July 2025).
